# p53 governs *t*elomere *r*egulation *f*eedback *too*, via TRF2

**DOI:** 10.18632/aging.100271

**Published:** 2011-01-25

**Authors:** Izumi Horikawa, Kaori Fujita, Curtis C. Harris

**Affiliations:** Laboratory of Human Carcinogenesis, National Cancer Institute, National Institutes of Health, Bethesda, MD 20892, USA

**Keywords:** telomere uncapping, p53, ubiquitin ligase, TRF2, feedback regulation

## Abstract

p53 takes critical part in a number of positive and negative feedback loops to regulate carcinogenesis, aging and other biological processes. Uncapped or dysfunctional telomeres are an endogenous DNA damage that activates ATM kinase (ataxia telangiectasia mutated) and then p53 to induce cellular senescence or apoptosis. Our recent study shows that p53, a downstream effector of the telomere damage signaling, also functions upstream of the telomere-capping protein complex by inhibiting one of its components, TRF2 (telomeric repeat binding factor 2). Since TRF2 inhibition leads to ATM activation, a novel positive feedback loop exists to amplify uncapped telomere-induced, p53-mediated cellular responses. Siah1 (seven in absentia homolog 1), a p53-inducible E3 ubiquitin ligase, plays a key role in this feedback regulation by targeting TRF2 for ubiquitination and proteasomal degradation. Biological significance and therapeutic implications of this study are discussed.

## Telomere DNA repeats and telomerase: Classical view of telomere biology

Chromosome ends have repetitive DNA sequences called telomere DNA repeats. The unit of the repeats is 5'-TTAGGG-3' in mammals, and the total length of the repeats reaches approximately 3 to 15 kilobase pairs (Kb) in humans, depending on cell types as well as other intrinsic and extrinsic factors. A major determinant of the telomere length in normal human differentiated cells (e.g., fibroblasts, which is a cell type most commonly used in cell culture experiments *in vitro*) is their replicative history, i.e., the number of cell divisions the cells have undergone [1]. Because of the inability of conventional DNA polymerases to replicate the very end of linear DNA molecules (so-called “end replication problem”), these normal cells experience a progressive decline in telomere length, as an indicator of “cellular aging”, in a cell division number-dependent manner. The presence of critically eroded telomeres in these cells is associated with the end of their replicative lifespan, a permanent growth arrest state called “replicative senescence” [1]. In contrast, germ cells, stem cells and cancer cells have a mechanism of synthesizing telomere DNA repeats to compensate the cell division-dependent telomere attrition, thereby bypassing the replicative senescence and being capable of self-replicating indefinitely [2]. Although ~10% of human cancers use a recombination-based mechanism (i.e., alternative lengthening of telomeres, ALT) [2], the major mechanism for telomere maintenance in human cells is the activation of a telomere G-rich strand-synthesizing enzyme, telomerase, which consists of the catalytic protein subunit (human telomerase reverse transcriptase, hTERT), the RNA component (hTER) containing a C-rich template region, and other associated proteins [3]. During a transition from normal mortal cells to immortalized, transformed cells, the transcriptional activation of the *hTERT* gene is a limiting step on which various cellular and viral oncogenic mechanisms act [4], including p53 inactivation [5]. Consistently, an ectopic expression of *hTERT* could lead to immortalization of normal human cells [6], and inhibition of *hTERT* in immortalized cancer cells could induce them to undergo senescence [7]. All of these findings accumulated from 1980's to late 1990's have supported “telomere hypothesis of cellular aging” to explain replicative senescence and immortalization of human cells.

## Telomeres as a DNA-multiprotein complex: Specific structure and functions

Since mid-late 1990's a growing body of evidence has established that not only telomere length but also its specific DNA structure is a key factor for normal telomere functions, and that a number of proteins associated with telomere DNA repeats play physiological roles at telomeres [8]. Telomere DNA was revealed to form a lariat structure, which is named “t-loop”, hiding chromosome ends into the structure and thus preventing them from undergoing illegitimate degradation or fusion events and from activating unwanted DNA damage signaling [9]. For the formation of this t-loop structure, a single-stranded, G-rich 3'-overhang at the extreme end of telomere DNA repeats plays an essential role by invading the double-stranded part of the repeats. The coordinated synthesis and processing of C-rich strand are required for the generation of 3'-overhang and thus essential for the telomere-specific DNA structure [10].

An increasing number of proteins have been shown to interact with telomere DNA repeats. Six of them (TRF1, TRF2, POT1, TIN2, TPP1 and RAP1) form a single telomere DNA-interacting complex, named “shelterin” [8]. TRF1 and TRF2 directly bind the double-stranded telomere repeats, POT1 directly binds to the single-stranded 3'-overhang, and the other three components interconnect these telomere-binding components. The shelterin complex is indispensable for the formation and maintenance of the telomere-specific DNA structure described above, as well as for distinguished recognition of telomeres from broken DNA ends. Specifically, TRF2 has an activity to enhance the t-loop formation [11] and can prevent ATM (ataxia telangiectasia mutated) kinase from initiating the DNA damage signaling at functional telomeres [12]. TRF2 interacts with ATM and inhibits its autophosphorylation critical for activation [13]. POT1 governs the integrity of telomere DNA ends at both G-rich 3'-overhang and C-rich 5'-recessed strand [14], as well as inhibits another DNA damage signaling kinase ATR (ATM- and Rad3-related) [12].

Taken all these findings together, we now recognize telomeres as a DNA-multiprotein complex with specific structure and functions, rather than only as an end of linear DNA molecules, and the concept of “telomere capping” has been established. The “capped” state of telomeres is primarily attributed to the t-loop structure of telomere DNA repeats and the recruitment and function of the shelterin complex [8,9]. Functional inhibition or knockdown of the shelterin components such as TRF2 and POT1 thus induced the “uncapped”, or dysfunctional, state of telomeres, which was characterized by loss of 3'-overhang, telomere fusion-induced chromosome instability and activated DNA damage signaling [15-17]. The classical view of telomere biology described above, however, still stands together with the updated view: either telomeres that are long enough (e.g., in normal, non-senescent cells) or activated telomerase (e.g., in cancer cells), or both (e.g., in germ cells and stem cells) could contribute to the strength and stable maintenance of the capped state of telomeres [18]. Long telomeres could provide a platform for enhanced recruitment of telomere-binding proteins (e.g., TRF2) and a high efficiency of t-loop formation in the absence of telomerase activity. The ability of telomerase to synthesize G-rich telomere DNA strand could generate 3'-overhang sufficient for short telomeres to form a t-loop. Consistently, the presence of neither long telomeres nor telomerase activity (for example, critically short telomeres at the end of replicative lifespan in telomerase-negative normal cells [19], and telomerase inhibition in cancer cells with short telomeres [20]) causes the telomere uncapping-associated phenotypes similar to those induced by inhibition of the shelterin components.

## p53 as a downstream effector of the telomere damage signaling

The tumor suppressor protein p53 is activated by endogenous or exogenous DNA damage and other cellular stresses through its posttranslational modifications such as phosphorylation, acetylation and sumoylation [21]. The p53-induced cellular phenotypes include apoptotic cell death and cellular senescence, each of which functions as a tumor suppressor mechanism and may have a role in organismal aging [22]. Classically, along with the telomere hypothesis of cellular aging, inhibition of p53 allowed normal human fibroblasts to continue proliferating even with shorter telomeres than the threshold length, suggesting that p53 was indispensable for the onset of telomere-initiated replicative cellular senescence [1]. With the concept of telomere capping, we now define uncapped or dysfunctional telomeres, which are functionally synonymous with eroded telomeres at the end of the cellular replicative lifespan [18,19], as an endogenous DNA damage that physiologically activates p53. Two major signaling kinases that phosphorylate and thereby activate p53 are ATM and ATR, which, as described above, are controlled by two shelterin components TRF2 and POT1, respectively [12]. ATM and p53 mediate the telomere uncapping-induced apoptosis or cellular senescence (for example, apoptosis in lymphocytes [23] and cellular senescence in fibroblasts [24]), establishing the TRF2-ATM-p53 pathway as a downstream effector pathway of the DNA damage response initiated at uncapped telomeres. Although ATR may be less essential to telomere damage signaling in human cells with functional ATM and its downstream targets, it is still capable of inducing p53-dependent cellular senescence independently of ATM [25]. Thus, the POT1-ATR-p53 pathway can also signal the DNA damage response from uncapped telomeres.

## p53 represses TRF2 protein levels through a p53-inducible E3 ubiquitin ligase Siah1

Comparing the expression profiles of endogenous mRNA or proteins in normal human cells at proliferating phase (referred “young”) *versus* at replicative senescence has identified a number of senescence-associated genes and proteins. Our cell system consists of an isogenic pair of young and replicatively senescent human fibroblasts, in the latter of which p53 activation is evidenced by its phosphorylation at serine 15 residue and the upregulation of p53 target genes such as *p21^WAF1^*[26,27]. Among those identified by our previous studies using this cell system are: Δ133p53 and p53β (p53 isoforms) [26], POT1v5 (a POT1 isoform) [17], ING2 (a p53- and chromatin-interacting protein) [28], miR-34a (a p53-inducible microRNA) [26], and WNT16B (a member of WNT family of secreted proteins) [29]. Our recent study [27] adds TRF2 to the list of proteins whose expression levels are changed during replicative senescence. The expression levels of TRF2 protein were reduced in replicatively senescent fibroblasts, but TRF2 mRNA levels did not change, suggesting the regulation at a post-transcriptional or protein level. A series of experimental manipulation of p53 expression and activity, including spontaneous allelic loss, short hairpin RNA (shRNA)-mediated knockdown, dominant-negative inhibition, nutlin-3a activation and retroviral overexpression, all suggested that p53 represses TRF2 protein levels, again without a change in TRF2 mRNA expression.

Siah1 is an E3 ubiquitin ligase with a C3HC4-type RING finger domain. The Siah1 gene is transcriptionally induced by p53 [30]. Siah1 targets proteins containing a Myb DNA-binding domain [31], which is present in TRF2 [15], for proteasome-mediated degradation. We have also found that TRF2 is a ubiquitinated protein and is subject to proteasome-mediated degradation [27]. All of these findings prompted us to examine whether Siah1 is responsible for the p53-mediated repression of TRF2 at the protein level. In the replicative senescence with activated p53 and all the experimental settings described above, Siah1 expression levels (both mRNA and protein levels) were inversely correlated with TRF2 protein levels: TRF2 upregulation was coincident with Siah1 downregulation when p53 was lost or inhibited; and TRF2 downregulation was coincident with Siah1 upregulation when p53 was overexpressed or nutlin-3a-activated [27]. The shRNA knockdown of Siah1 stabilized TRF2 protein by extending its half-life and, most importantly, Siah1 was found to physically interact with and ubiquitinate TRF2 in a manner dependent on its RING finger domain [27]. These data identified Siah1 as an E3 ubiquitin ligase that directly links p53 activation to TRF2 degradation, for the first time revealing that p53, a well-known downstream effector of the damage signaling from uncapped telomeres, also functions upstream to regulate a component of the telomere capping protein complex. Knockdown of endogenous Siah1 expression [27], as well as overexpression of TRF2 [32], delayed the onset of replicative senescence in human fibroblasts, suggesting that Siah1 co-operates with the other p53 target genes (e.g., p21^WAF1^ and miR-34a) [26] in p53-regulated senescence.

## TRF2-ATM-p53 positive feedback loop

**Figure 1.**
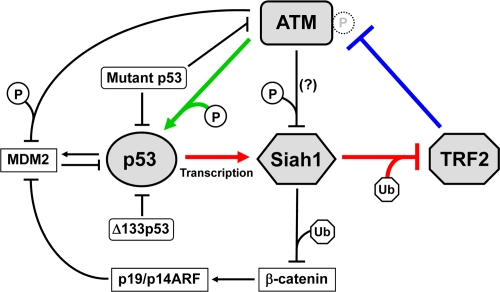
TRF2-ATM-p53 positive feedback loop. TRF2 interacts with ATM and inhibits its autophosphorylation and activation (blue line) [12,13]. ATM phosphorylates and activates p53 (green line) [23,24]. p53 transactivates Siah1, which targets TRF2 for ubiquitination and degradation (red lines) [27]. These regulatory mechanisms form a positive feedback loop. Mutant p53 abrogates this feedback loop through dominant-negative inhibition of wild-type p53 and a gain-of-function activity to inhibit ATM [48]. Δ133p53, a natural p53 isoform, also inhibits wild-type p53 [26]. The other factors that may functionally interact with this feedback loop (β-catenin, p19/p14ARF and MDM2) are also shown. P, phosphorylation. Ub, ubiquitination. Although ATM-mediated phosphorylation of Siah1 inhibits its interaction with a target protein (HIPK2) [36], it is unknown whether this mode of inhibition occurs for TRF2.

p53 is engaged in a number of positive and negative feedback loops to regulate carcinogenesis, aging and other biological processes [33]. p53 and MDM2, which is a p53-induced E3 ubiquitin ligase and targets p53 for ubiquitination and proteasomal degradation, form a minimum feedback unit that takes part in several larger and more complex feedback loops. These feedback loops also link the p53 signaling pathway to other signal transduction pathways, including those involving the cyclin E/cdk2 complex, the p38 MAP kinase, the WNT/β-catenin cascade and the PTEN/AKT cascade [33]. Given that TRF2 functions to negatively control ATM [12,13] (Figure 1, blue line) and that ATM phosphorylates and activates p53 [24] (Figure 1, green line), as described above, our finding of the p53-Siah1-TRF2 pathway [27] (Figure 1, red lines) uncovers a novel feedback loop involving these factors: This TRF2-ATM-p53 feedback loop contains two inhibitory regulations (TRF2 ⊣ ATM and Siah1 ⊣ TRF2), thus functioning as a positive feedback mechanism (we would prefer the word “positive feedback”, rather than “double-negative feedback”, to indicate the functional consequence of the whole loop). Once telomere uncapping occurs either endogenously at the end of cellular replicative lifespan or exogenously by reagents and conditions inducing telomere dysfunction, diminished amount and/or impaired activity of TRF2 results in ATM activation. ATM-mediated activation of p53 then transcriptionally activates Siah1, which ubiquitinates and degrades TRF2, leading to further decrease in TRF2 levels and thereby the maintenance and reinforcement of the uncapped state of telomeres.

The TRF2-ATM-p53 feedback loop amplifies telomere-initiated, p53-mediated DNA damage responses leading to the rapid induction of cellular senescence or apoptosis, which may prevent DNA damage-carrying cells from contributing to tumor formation.

Given the TRF2-ATM-p53 positive feedback loop, it is interesting to hypothesize that non-telomeric DNA damage and cellular stresses activating ATM and/or p53 may induce telomere uncapping through the repression of TRF2 protein levels. Consistent with this hypothesis, our preliminary data showed that doxorubicin-induced global DNA damage resulted in ubiquitination and downregulation of TRF2 [27]. It should also be noted that the TRF2-ATM-p53 positive feedback loop can functionally interact with the previously identified feedback regulations (Figure 1, black lines). β-catenin, another degradation target of Siah1 [30], is known to activate p19/p14ARF, which inhibits MDM2 and thereby activates p53 [34]. This regulatory circuit may act as a negative feedback element for the TRF2-ATM-p53 positive feedback loop. While ATM can also activate p53 indirectly through the phosphorylation and inhibition of MDM2 [35], ATM-mediated phos-phorylation of Siah1 may negatively affect its activity [36]. These co-operating and apparently opposing mechanisms are likely to add further strength and complexity to the DNA damage signaling from uncapped telomeres.

## Perspectives

Our identification of the p53- and Siah1-mediated regulation of TRF2 largely depended on *in vitro* experiments using normal human fibroblasts in culture. However, there is evidence that this regulation also exists *in vivo*. Human colon adenomas, a premalignant tumor in which p53-mediated cellular senescence is pathologically induced *in vivo*[37,38], were shown to express higher levels of Siah1 and lower levels of TRF2 than normal non-senescent tissues [27], recapitulating the expression profile observed in senescent fibroblasts *in vitro*. Further studies will investigate the role of the TRF2-ATM-p53 feedback regulation in *in vivo* physiological senescence, aging-associated genome instability and organismal aging in humans.

An attribution of cellular senescence, or aging at the cellular level, to aging at the organ and organismal levels can be explained by an aging-associated decline in stem cell function [39-41]. Accumulated DNA damage at telomeric and non-telomeric loci in tissue-specific stem cells during aging could cause cellular senescence or apoptosis in those cells, leading to reduced tissue regeneration, impaired organ function and homeostasis, and eventually organismal senescence. Since a growing body of evidence suggests that p53 activity and telomere integrity are critical to the regulation of self-renewing and differentiating properties of embryonic and adult stem cells [39,42-44], the TRF2-ATM-p53 positive feedback loop should have significant relevance to stem cell biology and stem cell-based medicine.

*p53* is the most frequently inactivated gene in human cancer [45,46], and therefore p53 replacement or restoration therapy is a therapeutic strategy against cancer. Telomeres have also been a promising target for cancer therapy: telomerase inhibitors can induce short telomeres in cancer cells to be uncapped [18]; and G-quadruplex ligands (e.g., telomestatin) reduce the amounts of G-rich 3'-overhang and remove POT1 and TRF2 from telomeric DNA, resulting in telomere uncapping [20,47]. We now know that these p53-based and telomere-based cancer therapies can co-operate in a single regulatory loop, namely the TRF2-ATM-p53 positive feedback loop. We expect that our findings will be experimental basis for combined application of p53-based therapy, telomere-based therapy and other strategies involving the associated factors such as β-catenin, p19/p14ARF and MDM2.

Some p53 missense mutations not only inhibit wild-type p53 activity but also gain a function that wild-type p53 does not have. One of such gain-of-function activities of mutant p53 is to inhibit ATM [48], which allows cancer cells to abrogate the TRF2-ATM-p53 feedback regulation in a more active manner than simply losing or inactivating wild-type p53. It will be of therapeutic interest to investigate whether small molecules that restore wild-type function to mutant p53 (e.g., CP-31398 and PRIMA-1) [49,50] can restore the TRF2-ATM-p53 positive feedback regulation in cancers with *p53* mutations. In cancers with wild-type *p53*, the expression of Δ133p53, a natural p53 isoform that inhibits wild-type p53 activity, is frequently elevated and thus Δ133p53 can be a therapeutic target [26]. Restoration of wild-type p53 activity by inhibition of Δ133p53 and the resulting degradation of TRF2 [26,27] may cause telomere uncapping-induced senescence or apoptosis through the TRF2-ATM-p53 positive feedback loop in cancers with wild-type *p53*.

## Conclusion

p53 has long been linked to the regulation of telomeres. In the classical view of telomere length regulation, p53 was a mediator of telomere-induced replicative senescence, as well as a negative regulator of telomerase via hTERT inhibition. Upon identification of the telomere-capping shelterin complex, p53 has been established as a downstream effector of the DNA damage signaling emerging from a specific shelterin component at uncapped telomeres. With evidence that p53 directly controls the shelterin component in a feedback manner, much attention to p53 continues to come from basic research on telomere biology, as well as from translational and clinical research aiming at telomere-based therapies.
